# Zinc in Early Life: A Key Element in the Fetus and Preterm Neonate

**DOI:** 10.3390/nu7125542

**Published:** 2015-12-11

**Authors:** Gianluca Terrin, Roberto Berni Canani, Maria Di Chiara, Andrea Pietravalle, Vincenzo Aleandri, Francesca Conte, Mario De Curtis

**Affiliations:** 1 Department of Gynecology-Obstetrics, University of Rome La Sapienza, Rome 00186, Italy; mariadc91@hotmail.it (M.D.C.); apietravalle@gmail.com (A.P.); vincenzo.aleandri@uniroma1.it (V.A.); 2 Department of Translational Medicine, University of Naples Federico II, Napoli 80138, Italy; berni@unina.it; 3 Research Center on Evaluation of Quality in Medicine—CEQUAM, University of Rome La Sapienza, Rome 00186, Italy; 4 Department of Pediatrics, University of Rome La Sapienza, Rome 00186, Italy; frconte2000@yahoo.it (F.C.); mario.decurtis@uniroma1.it (M.D.C.)

**Keywords:** micronutrients, neonate, newborn, fetus, low birth weight, growth, dermatitis, Necrotizing enterocolitis

## Abstract

Zinc is a key element for growth and development. In this narrative review, we focus on the role of dietary zinc in early life (including embryo, fetus and preterm neonate), analyzing consequences of zinc deficiency and adequacy of current recommendations on dietary zinc. We performed a systematic search of articles on the role of zinc in early life. We selected and analyzed 81 studies. Results of this analysis showed that preservation of zinc balance is of critical importance for the avoidance of possible consequences of low zinc levels on pre- and post-natal life. Insufficient quantities of zinc during embryogenesis may influence the final phenotype of all organs. Maternal zinc restriction during pregnancy influences fetal growth, while adequate zinc supplementation during pregnancy may result in a reduction of the risk of preterm birth. Preterm neonates are at particular risk to develop zinc deficiency due to a combination of different factors: (i) low body stores due to reduced time for placental transfer of zinc; (ii) increased endogenous losses; and (iii) marginal intake. Early diagnosis of zinc deficiency, through the measurement of serum zinc concentrations, may be essential to avoid severe prenatal and postnatal consequences in these patients. Typical clinical manifestations of zinc deficiency are growth impairment and dermatitis. Increasing data suggest that moderate zinc deficiency may have significant subclinical effects, increasing the risk of several complications typical of preterm neonates (*i.e.*, necrotizing enterocolitis, chronic lung disease, and retinopathy), and that current recommended intakes should be revised to meet zinc requirements of extremely preterm neonates. Future studies evaluating the adequacy of current recommendations are advocated.

## 1. Introduction

**Figure 1:**
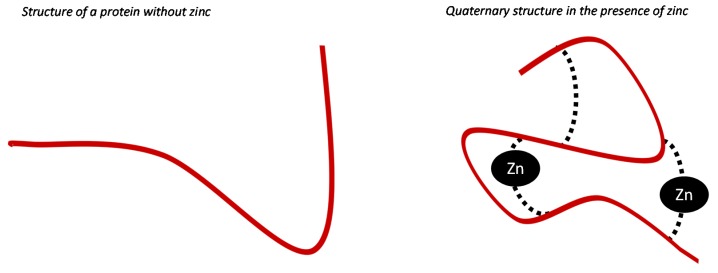
Zinc role in stabilizing protein quaternary structure [2].

Zinc is one of the most abundant trace elements in humans [1]. Zinc is functional for the activity of a number of proteins (*i.e.*, enzymes, membrane proteins, gene-regulatory proteins, and hormonal receptors) involved in most major metabolic pathways [2]. Zinc interacts with proteins in different ways: (i) by promoting enzymatic processes; (ii) by maintaining quaternary structure stability (Figure 1) [2]; or (iii) by favoring interactions with other molecules (*i.e.*, proteins, nucleic acids) [3]. The six enzyme classes established by the International Union of Biochemistry Enzymes, *i.e.*, oxidoreductases, transferases, hydrolases, lysases, isomerases and ligases, all require zinc [1]. During the enzymatic processes, zinc may have (1) a catalytic role (it has roles directly in catalytic processes), (2) a coactive role (by enhancing or diminishing catalytic functions) and a structural role (it is required for quaternary structure stability of enzymes) [4]. Metallothioneins (MTs) are the most abundant cysteine-rich proteins containing zinc [5]. These proteins have an important antioxidant activity and stabilize cell membranes only in the presence of zinc [6]. Zinc is also required for the DNA binding proteins involved in the regulation of gene expression [7,8,9]. The glucocorticoid and estrogen receptors are examples of zinc hormonal receptor proteins. They are members of a multigene family that includes receptors for thyroid hormone, retinoic acid, and vitamin D [10]. The DNA binding domain of these proteins contains zinc. Removal of this element yields hormonal receptors that do not bind DNA when activated by glucocorticoids or estrogens.

**Figure 2:**
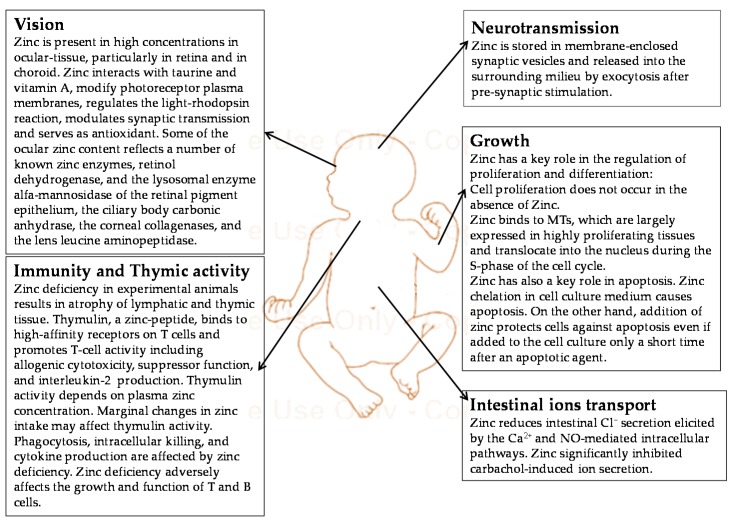
Zinc functions in early life [11,12,13,14,15,16,17].

The characteristic of the zinc is, certainly, its even distribution throughout the body. Functionally, zinc participates in cell division and growth, intestinal electrolyte absorption, neurotransmission, immune response, thymus activity, and vision (Figure 2) [11,12,13,14,15,16,17]. Consequently, numerous functions in humans are affected by zinc deficiency, particularly during periods of increased metabolism. However, clinical manifestations become evident only with severe deficiency; dermatitis, diarrhea, neurological disorders, growth failure, infections and delayed tissue healing following injuries are the most frequent clinical consequences of zinc deficiency. In early life, zinc deficiency may affect embryogenesis and may influence duration of pregnancy. After birth, a major factor associated with the development of zinc deficiency is its inadequate intake. Additional exacerbating factors include high physiological requirements, excessive losses by pathological conditions, intestinal failure and treatment with some drugs [18]. With some degree of variability, as with many other nutrients, human do not have functional reserves or body stores of available zinc, except neonates born at term, who may be able to draw on the hepatic zinc accumulated during the entire gestational period [19]. These aspects are of particular importance for preterm neonates. In this population, high requirements for growth and environmental injuries, physiological intestinal insufficiency and frequent use of antibiotics, significantly increase the risk of zinc deficiency. In addition, preterm birth reduces the duration of pregnancy and thus the amount of hepatic stores available during periods of reduced zinc intake.

Consequently, preservation of a positive zinc balance in the mother during pregnancy and lactation, and in neonates, is of critical importance in early life for the possible consequences on health, growth and development.

Starting from these considerations, we carried out a narrative review, analyzing current literature, with the subsequent aims to:

1. Explore the role of zinc in early life including embryogenesis, fetal and neonatal life.
2. Analyze the criteria used for the diagnosis of zinc deficiency in preterm neonates.
3. Investigate the consequences of zinc deficiency in preterm neonates.
4. Evaluate the adequacy of current recommendations on zinc for preterm neonates.
5. Suggest new possible research perspectives on the use of zinc in early life.

## 2. Evidence Selection Method

We performed a systematic search of articles on the role of zinc in embryogenesis, during fetal life and in babies born prematurely. We conducted an electronic search in MEDLINE using the following medical subject headings and terms: “zinc” associated with “embryogenesis”, “fetus”, “preterm neonate” or “preterm newborn”. In addition, we made a manual search of the reference lists of all eligible articles. We limited the search to clinical trials, case reports and reviews in human and animal model, in English language. We selected and analyzed 81 studies (Table 1) and report the results here in a narrative review.

**Table 1:** Evidence selection.

Medical Subject Headings and Terms	Zinc AND Embryogenesis	Zinc AND Fetus	Zinc AND Preterm Neonate or Zinc AND Preterm Newborn
Eligible articles	196	100	155
Excluded articles (reasons)	188 (unrelated articles)	86 (unrelated articles)	96 (unrelated articles)
Selected articles, *n*	**8**	**14**	**59**
-Human	**4**	**11**	**55**
-Animal	**4**	**3**	**4**

## 3. Function of Zinc during Fetal and Neonatal Life

### 3.1. The Role of Zinc in Embryogenesis

An adequate supply of maternal zinc is essential for embryogenesis. A major role of zinc is the regulation of chromatin structure and function and, thus, the expression of genes essential for embryogenesis [20]. Consequently, insufficient quantities of zinc during embryogenesis may influence the final phenotype of all organs [21]. Maternal zinc deprivation increases the risk of fetal mortality, growth retardation and malformations, including neural tube defects as demonstrated in clinical trials [22,23,24]. The vast majority of the evidence on the role of zinc in embryogenesis derives from the observation of the phenomenological effects of its deficiency in animal models rather than in human pregnancies (Table 2) [25,26,27,28,29,30,31,32]. Particular attention should be given to alcohol intake during pregnancy. Alcohol alters zinc metabolism and induces severe consequences on the fetus [33,34,35,36,37,38,39] Many of the clinical manifestation typical of the alcoholic fetal syndrome may be specifically related to zinc deficiency [33,34,35,36,37,38,39].

**Table 2:** The role of zinc in embryogenesis. Evidence from animal and human studies.

A. Evidence from Experimental Animal Model.					
Study	Model	Study Design	Main Results
Hurley *et al.*, 1969 [25]	Pregnant rats	Severe zinc deficiency induced by the use of a diet containing isolated soybean protein (treated with a chelating agent). Controls fed with zinc-supplemented diet	98% of full-term fetuses with congenital malformations of the tail (72%), finger (64%), lungs (54%), palate (42%), brain (47%), eye (42%), feet (38%), urogenital tract (21%)
Hicory *et al.*, 1979 [26]	Pregnant rats	Eighteen rats fed with zinc deficient diet and 18 fed with zinc supplemented diet during pregnancy	Malformations of the trunk and limbs in fetuses of zinc deficient mothers
Rogers *et al.*, 1985 [27]	Long-Evans hooded pregnant rats and fetuses	Determination of teratogenicity of maternal Zn deficiency in the Long-Evans hooded rat, examining the effects of Zn deficiency on Zn, Fe, and Cu concentrations in maternal and fetal tissues. Evaluation of the effects of Zn deficiency on the risk of abdominal and skeletal malformations	All fetuses presented malformations when zinc was supplemented at low doses
Falchuk *et al.*, 2001 [28]	Frog embryos	Deprivation of zinc in embryos to evaluate the effects on metallo-proteins activity and on organ formation and development	Agenesis of dorsal organs (including brain, eyes and spinal cord) in embryos developed in the absence of zinc.
**B. Evidence from Clinical Studies in Human.**					
**Study**	**Population**	**Study Design**	**Results**
Velie *et al.*, 1999 [29]	Mothers of infants with neural tube defect (NTD) compared with mothers of healthy neonates (controls)	Retrospective study on pre-conceptional use of vitamin, mineral, and food supplements, by filling a specific questionnaire	Risk of NTDs decreased with the increase in maternal pre-conceptional zinc intake
Cengiz *et al.*, 2004 [30]	Mothers of infants with neural tube defect diagnosed in the second trimester of gestation compared with mothers of healthy neonates (controls)	Case-control study to investigate the relationship between maternal micronutrient serum level (including zinc) and NTD occurrence in neonates	No strict correlation between zinc concentrations and NTD
Zeyreks *et al.*, 2009 [31]	Mothers of infants with neural tube defect (NTD) compared with mothers of healthy neonates (controls)	Case-control study to investigate the relation between cord blood and maternal micronutrient serum levels of (including zinc) and NTD occurrence in neonates	The mean maternal serum zinc level in mothers of neonates with NTD was significantly lower than those of controls
Dey *et al.*, 2010 [32]	Mothers of infants with neural tube defects (NTD) compared with mothers of healthy neonates (controls).	Hospital-based case-control study conducted with the objective of finding the relationship between serum zinc levels in newborns and their mothers and NTDs in a Bangladeshi population	NTD were more likely in subjects born from mothers with lower serum zinc level

### 3.2. Metabolism of Zinc in Fetal Life

Most fetal zinc accretion occurs after the 24th week of gestation [40]. As gestation progresses, fetal zinc concentrations constantly increase [3]. Zinc is stored in the fetal liver, [3] and transport and accumulation of zinc in the liver is mediated by MTs. Induction of hepatic MTs activities results in hepatic zinc accumulation.

In the last trimester, the mother transfers to the fetus up to 1.5 mg/Kg of zinc every day [41].

Placental transfer of zinc to the fetus is mediated mainly by an endocytic mechanism [42]. Affinity for zinc by the placenta does not vary with gestational age or with low maternal plasma zinc concentrations. Zinc deficiency in the fetus is observed only in the presence of severe maternal zinc deficiency, because placental transfer of this element is an active process and fetal zinc concentrations are maintained constantly higher than maternal levels [41]. On the other hand, the absence of a ready mechanism for adjusting possible fetal zinc deprivation reduces the effects of maternal zinc supplementation during pregnancy.

Evidence from rodent models and humans suggests that maternal zinc restriction during pregnancy influences fetal growth and health [25]. In humans, conflicting results have been obtained when this relation has been explored. A recent Cochrane meta-analysis, including 21 randomized controlled trials involving over 17,000 women, demonstrated that maternal zinc supplementation during pregnancy resulted in a reduction in preterm birth (risk ratio 0.86, 95% confidence interval 0.76–0.97) [43]. No clear benefit of zinc supplementation was demonstrated on many other maternal and neonatal outcomes, except for induction of labor in a single trial. On basis of their results, the authors suggest that since the preterm association could well reflect poor nutrition, studies to address ways of improving the overall nutritional status of populations in impoverished areas, rather than focusing on micronutrient and or zinc supplementation in isolation, should be considered as priority. However, many important aspects should be considered during the interpretation of the results of meta-analyses. The evidence for reduction in preterm birth was primarily demonstrated in trials involving low-income women. In some of these trials, women were also given other micronutrients (*i.e.*, iron, folate, vitamins or a combination of these). Risk of bias was high or unclear for many studies included in the analysis. Finally, the effects of maternal zinc supplementation on neonatal morbidities and long-term growth and neurodevelopment were investigated only in a small number of trials, reporting conflicting results [43]. However, consistent evidence has shown that maternal zinc supplementation starting in the second trimester and discontinued at delivery is associated with decreased infectious diseases during the first six months of infancy, presumably as a consequence of a positive effect of zinc on the immune response in early life [44].

Further studies may be useful to establish whether isolated zinc deficit may have consequences on otherwise well-nourished women and whether specific supplementation, in these cases, could improve fetal and neonatal short and long-term outcomes.

Specific diet regimens may increase the risk of zinc deficiency in women [45,46,47]. Table 3 reports different risks deriving from low zinc intake in women, according to diet regimen [45,46,47]. The current understanding of zinc homeostasis indicates that the primary determinants of zinc absorption are the amounts of ingested phytate, other than zinc [45,46,47]. High consumption of phytate reduces zinc absorption. The diet regimen should be monitored in pregnant women, to reduce the risk of zinc deficiency.

**Table 3:** Risk magnitude of zinc deficiency during pregnancy according to maternal diet [45,46,47].

Risk of Zinc Deficiency	Diet Characteristics
Low	Adequate protein content mainly from non-vegetable sources (*i.e.*, meat or fish), low cereals intake (phytate intake <500 mg/day)
Moderate	Mixed diet containing animal or fish protein, vegetarian or vegan diet not based on cereal or flours (phytate intake of 500–1500 mg/day)
High	Low animal protein intake, high unrefined, unfermented and ungerminated * cereals intake (phytate intake >1500 mg/day)

Note: * Germination of cereal grains or fermentation (e.g., leavening) of many flours can reduce antagonistic potency of phytates on zinc absorption.

## 4. Neonatal Zinc Metabolism

**Figure 3:**
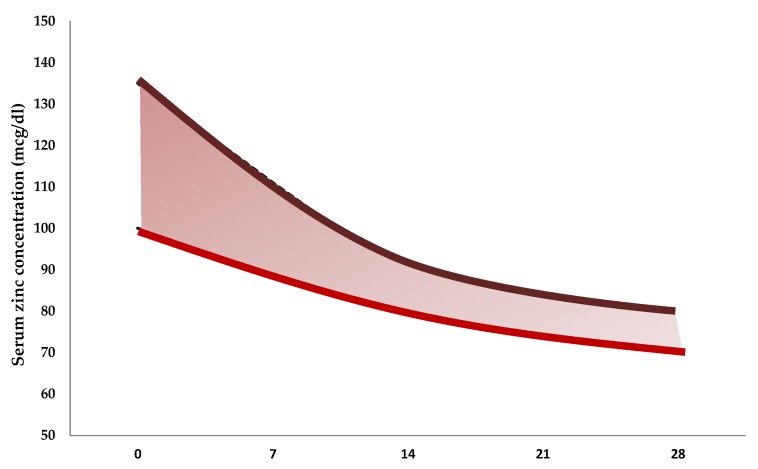
Serum zinc concentrations in preterm neonates by days of life [58,59,60,61].

Zinc deficiency has been well documented in hospitalized premature neonates [48]. The risk of zinc deficiency increases in preterm and small for gestational age (SGA) neonates or in case of intestinal failure [49]. The high risk of deficiency derives from a combination of three main factors: (i) low body stores due to reduced time for placental transfer of zinc; (ii) potentially marginal intake, and (iii) increased endogenous losses. Intestinal zinc absorption is not regulated by zinc status, rather, it is absorbed as a percentage of intake. However, in term babies, zinc absorption improves with decreasing serum zinc concentration and zinc stores are mobilized, enabling infants to avoid clinical deficiency. Zinc content in human milk varies considerably (0.7 to 1.6 mg/L) and declines with time [50,51,52,53,54]; while colostrum contains 8–12 mg/L, human milk at seven days of neonatal life contains 3–6 mg/L of zinc [50,51,52,53,54]. These values rapidly decrease at 1–3 mg/L at 1 month of life [50,51,52,53,54]. No clear differences in the zinc content of human milk were seen in women taking mineral supplements [50,51,52,53,54]. Preterm infant consuming 180 mL/kg per day of supplemented human milk would receive only 0.5–1 mg/kg per day of zinc [50,51,52,53,54]. Formula milk concentration of zinc is 1.5–6 mg/L [50,51,52,53,54]. Despite the greater concentration of zinc in formulas, net absorption was significantly improved with human milk as compared with formulas (60% *vs.* 20%) [50,51,52,53,54]. Absorption is slightly decreased in fortified preterm human milk as a percentage, although net absorption increases [50,51,52,53,54]. Endogenous fecal zinc losses are estimated to range between 50 and 150 mcg/kg per day [55,56]. The gastrointestinal tract is the major site of zinc losses, resulting from the secretion of endogenous zinc into the lumen and subsequent excretion in the feces. The amount of zinc excreted in the feces could be considered as a pool of rescue that could be reabsorbed and used according to host zinc status. However, the amount of zinc reabsorbed may also be influenced by the occurrence of pathological condition such as diarrhea and steatorrhea [55,56]. Urinary excretion of zinc is high in extremely preterm neonates (35 mcg/kg per day) in comparison with at term babies [55,57]. In the absence of adequate supplementation, serum zinc concentrations decline rapidly during the first months of life (Figure 3) [58,59,60,61].

A series of zinc transporter proteins (ZnT-1, ZnT-2, and ZnT-4) have been identified in the mammary gland [62]. In rare cases, the mammary gland may have a defect in the ZnT-4 gene, resulting in impaired secretion of zinc in breast milk. Breast milk zinc concentrations are below the normal range at all stages of lactation in these women [63]. Infants breast-fed by these mothers typically display the classic phenotype of acrodermatitis enteropathica already at approximately two months of postnatal age [63]. This syndrome has been described in term infants but is more likely to affect infants born preterm, suggesting an increased vulnerability of the preterm infant to clinically significant zinc deficiency syndromes [49]. Zinc concentrations in human milk may be influenced by vitamin A status, which regulates zinc transporters in the mammary gland (Table 4) [64,65,66,67,68,69,70,71,72,73,74]. In addition, the metabolism of zinc and vitamin A seems to be interrelated [72,74,75]. Severe vitamin A deficiency may reduce the absorption and lymphatic transport of zinc by altering synthesis of a zinc-dependent binding protein [75]. This aspect should be considered when zinc is supplemented in neonates receiving fortified human milk and multivitamin products containing vitamin A. These considerations suggest evaluating vitamin A supplementation, in addition to zinc supplementation, in neonates with evidence of zinc deficiency and in their mothers. However, the evidence of a strict zinc-vitamin A interaction in preterm neonates is still lacking.

**Table 4:** Mechanisms of the influence of other nutrients on zinc metabolism.

Nutrient	Mechanism
Proteins [64,65,66,67,68]	Protein is a major source of zinc, thus increased protein intake results in increased zinc intake High amounts of protein in enteral nutrition improve zinc absorption Casein in cow milk reduces zinc absorption
Lipids [69]	Fecal zinc increases in subjects with steatorrhea Medium-chain triglycerides improve zinc absorption
Copper [70]	Slight increase in copper intake does not interfere with zinc absorption if zinc intake is satisfactory. The effects of increased copper intake in subjects with low intake of zinc still remain to be defined.
Iron [71]	Iron administered at high doses (*i.e.*, iron-zinc ratio of 25:1 molar) reduce zinc absorption. Duration of iron supplementation does not affect zinc status
Vitamin A [72]	Severe vitamin A deficiency may reduce absorption and lymphatic transport of zinc by altering synthesis of zinc-dependent protein
Folic acid [73]	Supplementation with folate may impair zinc absorption by insoluble chelate formation

## 5. Diagnosis of Zinc Deficiency in Preterm Neonate

Accurate biomarkers of zinc status are still lacking. Measurement of serum zinc concentrations remains the best—albeit imperfect—marker to identify zinc deficiency (Table 5) [76,77,78,79]. Based on the data obtained from cord blood at birth, it is reasonable to identify a condition of zinc deficiency with serum concentrations at birth below 55 mcg/dL (8.4 mcmol/L) [80]. Serum zinc levels measured in cord blood at birth are higher in preterm compared with at term neonates (Table 6) [81,82,83,84,85,86]. Figure 3 depicts serum levels of zinc in preterm neonates in the first 28 days of life [58,59,60,61]. In preterm neonates, serum zinc levels rapidly decline during the first month of life, so that, at post-conceptional age of 40 weeks, serum zinc concentrations may be lower in preterm neonates compared with term neonates [58,59,60,61].

Typical clinical manifestations are commonly observed only for conditions of severe zinc deficiency. Increasing data suggest significant subclinical effects of a moderate zinc deficiency in preterm neonate [87]. Thus, an increase in zinc supplementation should be considered in all neonates with reduced serum zinc concentrations.

**Table 5:** Diagnostic tools for the diagnosis of zinc deficiency.

Biologic Samples Used for Zinc Concentrations Measurement	Characteristics	Limitations on the Use in Preterm Neonate
Serum or plasma [76]	It is the only biochemical indicator recommended by WHO to assess zinc status. Levels vary according to zinc intakes It may be used to predict response to zinc supplementation It is readily available as an early marker of severe deficit	Adventitious zinc can easily be added to samples by environmental exposure and inappropriate sample handling
		Zinc is released from hemolysed red blood cells into the serum.
		Low specificity (serum zinc concentrations decrease with a number of conditions such as infection, trauma, stress, steroid use, metabolic redistribution of zinc from the plasma to the tissues, concurrent nutrient deficiency).
		Starvation can induce the release of zinc in the circulation.
		The time of the day when blood samples are drawn has a significant effect on serum zinc concentrations (serum zinc is higher in morning samples than in afternoon or evening samples)
Intracellular concentrations (erythrocytes, platelets, leucocytes) [76,77]	Provides information on zinc status over a longer time period (independent of serum turn-over).	Absence of standardization and reference values in neonates
		Large volumes of blood required for the assay
		Sophisticated technology useful to isolate cells
Metalloenzymes [78]	Rapid response to zinc supplementation	No data on diagnostic accuracy in preterm neonates.
Hair [79]	Provides information on zinc status over a longer time period Easy to collect	Variability with age, sex, season, hair growth rate, and hair color
		No standardized methods for collection, washing, and analysis of hair samples in neonates

**Table 6:** Zinc levels in neonatal cord blood at birth by gestational age and birth weight.

Reference	Number of Neonates	Gestational Age at Birth, Weeks	Birth Weight, g	Mean Values ± Standard Deviation (µg/dL)
Jeswani *et al.*, 1991 [81]	25	<37	1790 ± 380	94 ± 18
	25	>37	2800 ± 200	129 ± 14
	10	>37	1880 ± 150	112 ± 9
Wasowicz *et al.*, 1993 [82]	51	>37		81 ± 24
	51	<37		93 ± 12
	23		1500–2499	85 ± 13
	41		2500–4750	81 ± 27
	13	24–36		92 ± 12
	15	37–38		87 ± 33
	36	39–41		78 ± 19
Iqbal *et al.*, 2001 [83]	3	28–33		90 ± 47
	29	34–36		88 ± 30
	22	37–39		83 ± 39
	11	40–41		79 ± 24
	11		1000–1500	103 ± 37
	16		1600–2000	81 ± 25
	10		2100–2500	79 ± 29
	18		2600–3000	83 ± 43
	10		3100–4000	81 ± 14
Perveen *et al.*, 2002 [84]	11	24–28		116 ± 45
	11	29–33		94 ± 19
	9	34–37		89 ± 15
	11	38–42		87 ± 9
Galinier *et al.,* 2005 [85]	53	26–31		160 ± 27
	76	31–33		137 ± 30
	66	33–34		125 ± 23
	53	34–37		128 ± 18
	262	>37	3234 ± 358	123 ± 20
Tsuzuki *et al.*, 2013 [86]	14	36 ± 2	2388 ± 465	89 ± 14
	30	39 ± 1	3043 ± 321	86 ± 16

## 6. Zinc Deficiency and Neonatal Complications

Typical signs of zinc deficiency in newborns include dermatitis and growth impairment [88,89]. However, the zinc status in the first weeks of life may influence the occurrence of many other pathological conditions interfering with their crucial pathogenic steps.

### 6.1. Dermatitis

Rash is the most common clinical feature of zinc deficiency, often misdiagnosed as Candida, eczema, or impetigo [88,90]. In preterm infants, the characteristic skin changes may occur early in the anterior neck fold, with poorly marginated erythema in the depth of the fold, that becomes well demarcated and scaled within five days [80,88,90]. Deficient patients respond quickly to oral zinc supplementation, usually within one week [91]. Many of the skin lesions observed in preterm neonates after the first week of life may depend on a zinc deficiency [51,52,53,54,55,56,57,58,59,60,61,62,63,64,65,66,67,68,69,70,71,72,73,74,75,76,77,78,79,80,81,82,83,84,85,86,87,88,89,90,91,92]. Thus, evaluation of zinc status is recommended in the newborn with dermatologic manifestation that cannot be explained otherwise.

### 6.2. Growth Retardation

Zinc intake has been associated with growth outcomes in both term and preterm infants over the first 12 months [51,93]. It seems clear that very preterm infants, who have poor zinc stores and great need for growth, are at particular risk of zinc deficiency, as shown by Ram Kumar *et al.* [94]. Itabashi *et al.* demonstrated an increased risk of low serum zinc levels with higher weight gain [95]. This phenomenon could be due to the increased enzyme activity and protein synthesis associated with growth. Zinc is a key element in the synthesis of proteins essential for growth [96]. Neonates with improved growth have greater zinc requirements and, consequently, present a higher risk of nutritional deficiency. However, this hypothesis remains, at present, still uninvestigated. On the other hand, zinc deficiency may limit neonatal growth; thus, its diagnosis should be considered for any preterm infant who is not growing well despite an apparently adequate energy and macronutrient intake, who has not consistently received a zinc-fortified human milk or formula designed for preterm infants, who has sustained any gastrointestinal resection or insult, or is on diuretic therapy [50,51,97].

### 6.3. Necrotizing Enterocolitis

Necrotizing enterocolitis (NEC) is the most severe gastrointestinal emergency in neonates. In some intensive care units, NEC affects up to 10% of premature infants [98]. The pathogenic mechanisms of NEC are not yet completely understood. The combination of intestinal immaturity, hemodynamic instability, asphyxia, infections and impaired inflammatory response underlies the development of NEC [98]. Nutritional approach may significantly influence the risk of NEC [99]. Despite that the role of zinc in many intestinal functions and clinical conditions of children has been widely studied, the clinical effects of zinc on the neonatal intestine remain largely unexplored. Several experimental models and clinical evidences suggest a role for zinc in the pathogenesis of NEC [87,100,101]. Zinc modulates the expression of important inflammatory cytokines and their receptors in the intestine in models of colitis [102]. Subjects deficient in zinc showed a reduced immune response against pathogens [103]. Zinc also has trophic effects on intestinal mucosa and modulates intestinal permeability [104,105]. Finally, zinc deficiency worsens the extent of damage from asphyxiation due to the reduced enzymatic antioxidant activities [106]. However, no systematic study has been carried out so far on the possible role of zinc in the development of NEC. A multicenter trial on premature babies reported a significant reduction in NEC incidence in subjects receiving high doses of zinc [87]. Based on the above-mentioned evidence, it is reasonable to imagine a role for zinc in NEC prevention, and design specific studies in this field.

### 6.4. Neurologic Damage

Zinc regulates the expression of neurotrophic factors that reduce apoptosis subsequent to different types of insults and promote neuronal regeneration [107,108]. Very low birth weight babies (VLBW) are at high risk of hypoxic brain damage. Severity of brain damage is influenced by the activity of zinc-dependent metallothioneins [109]. In different models of hypoxia, brain lesions are directly correlated with zinc concentrations in experimental conditions [110]. Zinc seems essential also in the modulation of vascular tone at the cerebral level [111]. The reduced ability to control the tone of cerebral vessels is of critical importance in the development of hypoxic-ischemic injury and intraventricular hemorrhage in the preterm infant [108]. However, clinical trials on the role of zinc in the prevention of brain damage in preterm neonate are not available at the moment.

### 6.5. Bronchopulmonary Dysplasia

Despite significant improvement in neonatal care and the consequent reduction in mortality associated with respiratory complications, the incidence of bronchopulmonary dysplasia (BPD) has remained unchanged over the years [112]. In the last two decades, clinical presentation of BPD has varied significantly. New types of BPD, characterized by growth arrest of the respiratory tract or by destructive lesions, usually occur in extremely preterm infants (gestational age <28 weeks, birth weight <750 g), with little chance of survival until a few years ago [113]. In the new BPD, nutritional support is essential to promote the development of the respiratory tract and prevent further inflammatory damage deriving from mechanical ventilation and local or systemic infections [113]. For these reasons, prestigious scientific societies have recommended specific nutritional intakes for the prevention of bronchopulmonary dysplasia (BPD) [114]. The action of antioxidant enzymes is essential to reduce the oxidative damage induced by mechanical ventilation, and the remodeling action of metalloproteinase is essential in response to inflammatory damage [115]. Zinc promotes epithelial development, participates in the enzymatic reactions underlying the repair of tissue damage, protects against infection, and modulates the inflammatory response in the respiratory system [116]. Thus, it is reasonable to hypothesize a role of zinc in preventing bronchopulmonary dysplasia. However, clinical studies demonstrating a clear relationship between zinc and BPD are not yet available.

### 6.6. Infections

It has been clearly demonstrated that zinc deficiency increases risk and severity of infections by reducing immune response to pathogens [117]. Zinc is a crucial element of the immune response. The presence of zinc is essential to ensure the normal activity of enzymes, peptides and cytokines in the cells of immune system. This element stabilizes the membranes of immune cells by acting on the cytoskeleton and regulates apoptosis, diapedesis and recruitment of immune cells. A wealth of evidence and recent trials confirm the utility of zinc in the prevention and treatment of intestinal and extra intestinal infections [117,118]. However, there is still no data available on the effectiveness of zinc in preventing infectious diseases in preterm neonates.

### 6.7. Retinopathy of Prematurity

Retinopathy of prematurity (ROP) is a major cause of blindness worldwide [119]. ROP is induced by multiple pathogenic phases. Exposure to high oxygen concentrations induces the cessation of retinal vessel growth after premature birth, through the down-regulation of the expression of growth factors essential for adequate retinal development (*i.e.*, Vascular endothelial growth factor: VEGF) [117]. The vascular depletion of the retina associated with body growth induces a state of chronic hypoxia and exposes the retina to the continuous risk of ischemia, especially in critically ill neonates [120]. Oxidative damage and inflammation, deriving from frequent ischemia-reperfusion injury, leads to an abnormal proliferation of vessels and pathologic neovascularization [119]. Zinc is the most abundant trace metal in the retina and may serve a critical function as antioxidant in this tissue [20]. Zinc also induces synthesis of metallothioneins, zinc-ion-binding proteins with a wide range of functions, including defense against oxidative damage and inflammation induced by hypoxia [120]. The human eye is enriched in metallothioneins. These zinc-enzymes contribute to antioxidant mechanisms of defense in the retina as in other ocular tissues [121]. It is possible to hypothesize a role of zinc in the inhibition of neovascularization, not only by reducing oxidative damage but also by controlling the expression of growth factors (*i.e.*, VEGF). This issue may represent a fascinating field of research for future studies.

Despite these plausible assumptions, trials in neonates have failed to demonstrate the efficacy of antioxidants in preventing ROP. On the other hand, there is encouraging data from a trial on preterm neonates demonstrating a trend to a reduction in ROP in subjects receiving high doses of zinc [87].

## 7. Supplementation of Zinc

**Figure 4:**
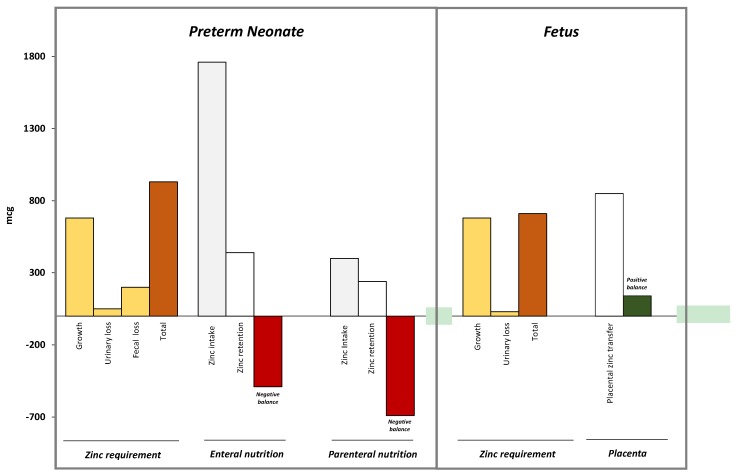
Zinc balance in fetal and neonatal life [132,133].

Preterm infants are generally believed to be in a negative zinc balance within the first 4–8 weeks of life, unless intakes are not bolstered by specific supplements [122]. Zinc could be provided through parenteral nutrition support, or fortified human milk, formulas designed for premature infants, or specific products containing zinc. Zinc-supplemented parenteral nutrition early in postnatal life, followed by enteral administration of zinc in adequate doses, should aim to be sufficient to meet zinc requirements also for extremely low birth weight infants (ELBW). Several indications for zinc requirements are available (Table 7) [123,124,125,126,127,128,129,130]. Interestingly, recommended intakes have progressively increased during the last decade. The recommended enteral intake ranges between 0.8 to 3 mg/Kg/day [3]. Zinc requirements for term infants are estimated to be 0.8 mg/day, whereas preterm neonates may require up to 3 mg/kg per day to achieve adequate zinc retention [3]. Less variability regarding intravenous supplementation may be found in the recommendations of different scientific societies and committees. Despite that only 60% of parenterally infused zinc is retained, different authors suggest parenteral supplementation with 350 mcg/kg per day [51,131]. Recent evidence supports the use of higher zinc doses to improve survival and reduce morbidity in VLBW neonates [87]. Higher zinc supplementation is also justified if we consider that in the last trimester of pregnancy the fetus receives up to 1 mg/Kg/day of zinc from the mother. Thus, to reach a similar intake, 0.5–0.8 mg/Kg/day or 4–5 mg/Kg/day zinc should be administered by the parenteral or oral route, respectively (Figure 4) [132,133]. However, further trials, including pharmacokinetic studies, are advocated before modifying current recommendations on the use of zinc.

Zinc requirement in preterm neonates is quite similar to that of fetuses with similar postconceptional age. However, a lower percentage of zinc administered in preterm neonates by enteral or parenteral nutrition is retained. Thus, if zinc is provided according to current recommendation (columns zinc intake), the final balance of zinc is negative, at variance with what happens during fetal life, when zinc is transferred by the placenta, directly in the fetal circulation, at a daily amount of about 900 mcg.

**Table 7:** Recommendations for zinc in neonates by enteral or parenteral route.

Institute/Scientific Societies/Academic Groups	Publication Year	Population of Neonates	Dose (mg/Kg/Day)
**Enteral route**			
The American Academy of Pediatrics Committee on nutrition [123]	1985	All	0.6
Committee on Nutrition of the Preterm Infant, European Society of Paediatric Gastroenterology and Nutrition [124]	1987	All	0.7–1.4
Zlotkin *et al.* [125]	1996	0–14 days of life	0.5–0.8
		>14 day of life	1
Klein *et al.* [126]	2002	Birth weight <1 Kg	2
		Birth weight 1–2 Kg	1.7
		Birth weight >2 Kg	1.3
Hambidge *et al.* [127]	2006	Birth weight <1 Kg	2.4
		Birth weight 1–2 Kg	2
		Birth weight 2–3.5 Kg	1.6
ESPGHAN Committee on Nutrition [128]	2010	Birth weight <1.8 Kg	1.1–2.0
Griffin *et al.* [129]	2013	Human milk feed	2.3–2.4
		Formula feed	1.8–2.4
**Parenteral route**			
American Society of Clinical Nutrition [130]	1988	All	0.4
Zlotkin *et al.* [125]	1996	Transitional period	0.15
		Stable period	0.4

## 8. Excessive Exposure to Zinc

Zinc supplementation is generally safe. However, an excess of zinc (>20 mg/Kg/day) may influence absorption and retention of other trace elements such as copper and vitamin A [50,74]. Zinc may induce copper deficiency by inhibiting the gastrointestinal absorption [134]. On the other hand, copper supplementation boosts the conjugation of zinc with large molecules and depletes the ratio of zinc coupled with smaller molecules, thereby suggesting an antagonism between the transport of these two elements [135]. Indeed, no cases of hypocupremia have been described in trials using zinc in neonates (with doses ranging from 2 to 10 mg/Kg/day) not even when copper was not supplemented.

Zinc status influences vitamin A absorption, transport and utilization [74]. Evidence of an effect of zinc intake on vitamin A status from animal experiments are inconclusive, mainly because of the use of inadequate control groups [136]. Randomized trials in children have failed to show a consistent effect of zinc supplementation on vitamin A status [137] and there is no such evidence in neonates. To the best of our knowledge, none of the other potential consequences of zinc excess, such as cytopenias or myeloneuropathy, has been reported in neonates.

## 9. Conclusions

Zinc plays a crucial role during the first phase of life, including embryogenesis and fetal life. Preterm neonates continue their “fetal” development in an extra-uterine environment. Consequently, zinc stores are not completed and its requirement increase in babies born prematurely. Considering the large number of roles played by zinc in early life, the consequences of zinc deficiency in preterm neonates are extremely variable and may be severe in many cases. A large percentage of preterm neonates is affected by mild to moderate subclinical zinc deficiency. Diagnosis of zinc deficiency, especially if of mild severity, is not easy due to its nonspecific features and to the lack of highly sensitive biomarkers. On the other hand, early diagnosis may be essential to avoid severe consequences of zinc deficiency. Clinical evaluation and research of the risk factors associated with the measurement of serum level of zinc is the most appropriate approach in the diagnosis of zinc deficiency. Dealing with the deficiency by zinc supplements is highly effective in neonates, with proven benefits on skin lesions and growth. Supplementation with adequate doses of zinc may also reduce the occurrence and severity of the morbidities typical of prematurity. However, current recommendations for zinc seem inadequate to meet the requirement of extremely preterm neonates and should be revised.

Despite the consistent number of articles on the role of zinc in early life, many interesting aspects remain to be clarified. Possible area of future research may be summarized in ten issues:

1. Study of the role of zinc in infertility and multiple abortions
2. Identification of diet and environmental determinants of zinc absorption in otherwise well-nourished pregnant women
3. Evaluation of zinc metabolism in neonates with alcoholic syndrome
4. Definition of determinants of zinc absorption in preterm neonates
5. Calculation of diagnostic power of zinc concentration assessment in cells and hairs, and of metalloenzyme in preterm neonates
6. Definition of modalities for zinc supplementation in preterm neonates (doses, administration route, duration of therapy)
7. Evaluation of the usefulness of individualized zinc supplementation in human milk feeding for preterm neonates
8. Study of the relation between zinc and vitamin A in preterm neonates
9. Study of the relation between zinc deficiency and occurrence of morbidities (*i.e.*, NEC, brain damage, BPD, infectious diseases, ROP) in preterm neonates.
10. Determination of zinc efficacy in reducing severe complications of prematurity and the study of the related mechanisms

When such data become available, the role of zinc in early life will be further clarified and the use of zinc in neonates could be optimized. Considering the critical role of zinc in neonatal life, research in this field is widely advocated.
